# Diagnostic Accuracy of Fetal Anterior Abdominal Wall Thickness as an Early Sonographic Sign for Diagnosing Gestational Diabetes

**DOI:** 10.7759/cureus.46538

**Published:** 2023-10-05

**Authors:** Muhammad Sharjeel Sabir, Muhammad Mujeeb Hassan, Moeena Malik, Rabia Saleem, Zartasha Tariq, Khurram Zohaib, Maham Javaid, Adnan Younas Malik, Amna Saleem

**Affiliations:** 1 Radiology, Holy Family Hospital Rawalpindi, Rawalpindi, PAK; 2 Internal Medicine, Rawalpindi Medical University, Rawalpindi, PAK; 3 Obstetrics and Gynecology, Rawalpindi Medical University, Rawalpindi, PAK; 4 General Medicine, Holy Family Hospital Rawalpindi, Rawalpindi, PAK; 5 Internal Medicine, Benazir Bhutto Hospital, Rawalpindi, PAK; 6 Internal Medicine, Allama Iqbal Medical College, Lahore, PAK; 7 Internal Medicine and Nephrology, District Headquarter Hospital, Rawalpindi, PAK; 8 Pediatrics and Child Health, Islamic International Medical College Trust (IIMCT)Pakistan Railways Hospital, Rawalpindi, PAK

**Keywords:** pregnant females, body mass index: bmi, fetal macrosomia, fetal ultrasound, diabetes gestational

## Abstract

Introduction

Traditionally, different fetal variable measurements are used in ultrasound to assess fetal growth. Ultrasound can detect abnormal fetal growth. Gestational diabetes mellitus (GDM) is linked to higher fetal obesity as early as 20 weeks of pregnancy. The amount of fetal adipose tissue may be measured by measuring the thickness of the anterior abdominal wall. Measuring the thickness of the fetus's anterior abdominal wall (AAWT) is a straightforward procedure that may be performed alongside standard abdominal circumference measurements.

Objectives

To check the diagnostic accuracy of fetal AAWT as an early sonographic sign for diagnosing GDM, keeping oral glucose tolerance test as the gold standard.

Study design

This research was conducted using a cross-sectional analysis.

Study place and duration

The study was conducted in the Radiology Department at Rawalpindi Medical University and Allied Hospitals from July 10, 2019 to January 9, 2020.

Materials and methods

Women between the ages of 18 and 45 who had a family history of type 2 diabetes and were at risk for developing GDM were recruited. Exclusions were made for diabetic women, those carrying multiples, and those with autoimmune diseases. The AAWT measurement of the fetus, which included the skin and subcutaneous tissue, was acquired using the traditional anterior cranial view, 2-3 cm lateral to cord insertion. Pregnant patients at risk for GDM underwent screening using an oral glucose tolerance test. Those exhibiting any two abnormal values were diagnosed with GDM.

Results

The overall sensitivity, specificity, positive predictive value, negative predictive value, and diagnostic accuracy of fetal AAWT as an early sonographic sign for diagnosing GDM, with the oral glucose tolerance test as the gold standard, were 93.14%, 82.65%, 84.82%, 92.05%, and 88.0%, respectively.

Conclusion

The study concludes that the diagnostic accuracy of fetal AAWT as an early sonographic indicator for identifying gestational diabetes is notably high.

## Introduction

Uncontrolled diabetes during pregnancy represents one of the most significant concerns due to its pervasive nature [1]. Gestational diabetes mellitus (GDM) encompasses any degree of glucose intolerance that is developed or initially diagnosed during pregnancy. GDM is characterized by high blood sugar levels caused by either a lack of insulin production or insulin resistance during pregnancy. This causes glucose levels in the blood to rise. Midway through or later in a pregnancy is often when symptoms of GDM first appear. Situations involving GDM are more prone to complications such as pre-eclampsia, depression, and the necessity for a Cesarean section (C-sec) [1-2]. Nguyen CL et al. found that 10.1% of pregnant women in East and Southeast Asia struggle with GDM [2]. Early and precise evaluation of fetal well-being and development is a cornerstone of obstetric care for patients with GDM. GDM is a frequent pregnancy condition linked to negative consequences for both the mother and the unborn child, such as an increased risk of type 2 diabetes and cardiovascular disease in the mother and an increased risk of macrosomia and obesity in the child [3]. Recent research has discovered that fetal overgrowth related to GDM begins before the condition is diagnosed, underscoring the urgent need for early detection of gestational glucose intolerance [4].

As a result, pre-GDM is a condition that must be carefully monitored, and women should be counseled prior to conception. Macrosomia and related complications are more probable in pregnancies where glycemic control is inadequate, which might occur later in pregnancy [3-4]. Additionally, perinatal mortality (encompassing stillbirths and neonatal deaths within the first week) and morbidity (such as neonatal hypoglycemia, macrosomia, low birth weight, and birth asphyxia) are approximately two to four times higher in women with diabetes compared to those without [5]. Ultrasound has traditionally relied on several separate measures of fetal variables to determine fetal development. Ultrasound is a reliable method for detecting fetal growth anomalies [3]. GDM is linked to higher fetal obesity as early as 20 weeks of pregnancy [4]. The amount of fetal adipose tissue may be measured by measuring the thickness of the anterior abdominal wall. Measuring the fetus's AAWT is a straightforward procedure that may be performed alongside standard abdominal circumference measurements [5]. Patients with GDM are more likely to have macrosomic or enormous babies for their gestational age [5]. A few studies have shown that this straightforward sonographic test may accurately predict fetal growth limitation and macrosomia in the general or diabetic population. Using a threshold of 2 mm for increased AAWT, Sinno SH et al. found that measuring the fetus's AAWT in mothers with GDM between the ages of 28 and 30 weeks had a sensitivity of 94.4% and a specificity of 14.6% [1]. However, several of these studies lacked necessary elements, such as a control group and adequate sample sizes. Fetal hyperinsulinemia is triggered by maternal hyperglycemia, especially in the second trimester [6].

When comparing mothers with and without GDM, the conventional biometric measures of fetuses (biparietal diameter, fetal abdominal circumference, femur length, and estimated fetal weight) show no change; however, fetal AAWT shows a substantial difference between pregnant women with and without GDM. We chose to conduct this research using ultrasound for diagnosing GDM because it is more affordable, widely accessible, less intrusive, and time-saving.

## Materials and methods

A total of 200 patients were enrolled in the study from July 10, 2019 to January 9, 2020, recruited in the Radiology Department in the Rawalpindi Medical University and Allied Hospitals. Patients were selected by non-probability, consecutive sampling. Written informed consent and a detailed history were taken from every patient before enrollment. Prior approval was sought from the hospital's ethical committee. Patients at risk for GDM presenting at 24-28 weeks of gestation were enrolled in the study. All patients underwent ultrasound as per protocol, followed by an oral glucose tolerance test. The Honda HS-2600 ultrasound system with the curved array transducer was used for the trans-abdominal examination. Radiologists with at least two years' experience, working in isolation and without knowledge of the patient's diagnosis, took all ultrasound readings. Fetal skin and subcutaneous tissue were included in the AAWT measurement, taken in the conventional anterior cervical (AC) perspective, 2-3 cm lateral to cord insertion. The AAWT was measured in millimeters using magnification at the AC level. The calipers were carefully positioned to measure the thickness of the skin on the anterior abdominal wall. Pregnant patients at risk for GDM were screened for GDM using an oral glucose tolerance test. Patients underwent testing of fasting plasma glucose levels. Then, 100 grams of oral glucose was administered, and again, plasma glucose levels were measured at 1-hour, 2-hour, and 3-hour intervals after oral glucose administration. Patients having any two abnormal values were labeled as suffering from GDM. GDM diagnosed based on the blood sugar levels were taken as the gold standard.

The inclusion criteria encompass pregnant patients between 24 to 28 weeks of gestation who are at risk of developing GDM, namely those with a family history of type 2 diabetes. The pertinent risk factors include a sedentary lifestyle (characterized by excessive sitting and minimal to no exercise), high blood pressure (a history of systolic blood pressure greater than 140 and diastolic greater than 90 after 20 weeks of gestation), obesity (BMI >30), and being between 24-28 weeks pregnant with at least two out of four abnormal plasma glucose levels. These levels are measured at fasting and at 1, 2, and 3 hours after per oral administration of 100 grams of glucose, with cut-off levels of 95 mg/dl or above for fasting, 180 mg/dl or above at 1 hour, 155 mg/dl or above at 2 hours, and 140 mg/dl or above at 3 hours, as specified by the American Diabetes Association. Exclusion criteria encompass patients diagnosed with diabetes before pregnancy, those carrying twins, and those with autoimmune diseases.

Statistical analysis

Data were recorded and analyzed using the SPSS v 22.0 (IBM Corp., Armonk, NY, USA) for Windows. Continuous data, such as age, gestational age, and anterior abdominal wall thickness, were presented as mean and SD. Categorical data, including the presence and absence of diabetes, hypertension, socioeconomic status, and level of education, were presented as frequency and percentage. A receiver operating characteristic (ROC) curve was plotted to determine whether AAWT could predict GDM in mothers. Effect modifiers, like maternal age, gestational age, hypertension, socioeconomic status, parity, and level of education, were controlled by post-stratifying diagnostic accuracy. From the ROC curve, an optimal cut-off for AAWT was determined that best established sensitivity and specificity. A 2 x 2 table was created, and sensitivity, specificity, positive predictive value, negative predictive value, and accuracy were determined. A p-value of <0.05 was considered statistically significant.

## Results

The age range in this study was from 18 to 45 years, with a mean age of 28.23 ± 5.23 years. A substantial majority of the patients, 157 (78.50%), were aged ≤35 years. Patient distribution according to parity is also discussed. The mean BMI was documented at 34.46 ± 2.67 kg/m^2, while the mean gestational age was recorded as 25.85 ± 1.37 weeks. Further distribution of patients, according to factors such as hypertension (HTN), socioeconomic status, and education, is elucidated in Table 1.

**Table 1:** Basic demographics of females (n=200). GA: Gestational age; HTN: Hypertension.

	Age (years)	No. of Patients	%age
Age	<35	157	78.50
	≥35	43	21.50
Parity	1-2	90	45.0
	>2	110	55.0
GA (weeks)	24-26	140	70.0
	27-28	60	30.0
HTN	Yes	43	21.50
	No	157	78.50
Socioeconomic status	Low	41	20.50
	Middle	115	57.50
	High	44	22.0
Education	Illiterate	80	40.0
	Literate	120	60.0

In the study, among the 112 patients who tested positive for fetal AAWT, 95 were confirmed as true positives for GDM. At the same time, 17 were false positives, exhibiting no GDM upon undergoing an oral glucose tolerance test. Overall sensitivity, specificity, positive predictive value, negative predictive value, and diagnostic accuracy of fetal AAWT as an early sonographic sign for diagnosing GDM, keeping oral glucose tolerance test as the gold standard, was 93.14%, 82.65%, 84.82%, 92.05%, and 88.0%, respectively. Stratification of diagnostic accuracy with respect to age group, GA, parity, HTN, socioeconomic status, and education is shown in Table 2.

**Table 2:** Diagnostic accuracy of fetal AAWT as an early sonographic indicator for diagnosing gestational diabetes, utilizing oral glucose tolerance test as the gold standard. HTN: Hypertension; SES: Socioeconomic status; AAWT: Anterior abdominal wall thickness.

	Sensitivity	Specificity	Positive Predictive Value	Negative Predictive Value	Diagnostic Accuracy
Overall	93.14%	82.65%	84.82%	92.05%	88.00%
Age <35 years (n=157)	93.59	81.01	82.95	92.76	87.26
Age >35 years (n=43)	91.67	89.47	91.67	89.47	90.70
Gestational age 24-26 weeks (n=140)	91.14	80.33	85.71	87.50	86.43
Age 27-28 weeks (n=60)	100	86.49	82.14	100	91.67
Parity 1-2 (n=90)	93.75	80.95	84.91	91.89	87.78
Parity >2 (n=110)	92.59	83.93	84.75	92.16	88.18
HTN (n=43)	94.74	95.83	94.74	95.83	95.35
HTN (n=157)	92.77	78.38	82.80	90.63	85.99
Low SES (n=41)	100	79.17	77.27	100	87.80
Middle SES (n=115)	90.48	84.62	87.69	88	87.83
High SES (n=44)	95.45	81.82	84	94.74	88.64
Illiterate women (n=80)	95	82.50	84.44	94.29	88.75
Literate women (n=120)	91.94	82.76	85.07	90.57	87.50

## Discussion

Early and precise evaluation of fetal well-being and development is the cornerstone of obstetric care for patients with GDM [6]. Traditional sonographic fetal development evaluation relies on the measurement of several biometric markers, including the biparietal diameter (BPD), femur length (FL), abdominal circumference (AC), and estimated fetal weight (EFW) [7-8]. The AC and the AAWT of the fetus are both straightforward measurements. In the general population or in pregnant women with diabetes, this straightforward sonographic assessment has been shown to accurately predict fetal growth limitation and macrosomia. However, several of these studies lacked important controls, had insufficient sample sizes, or used outdated diagnostic criteria for type 2 diabetes. Most of these randomized controlled studies also looked at pregnancies in their later third trimester. Fetal hyperinsulinemia is triggered by maternal hyperglycemia, especially in the second trimester. Macrosomic newborns and those born to moms with diabetes have been reported to have accelerated fetal development as early as the 18th week of pregnancy [9-11]. However, while screening for GDM in the early second trimester, there is no difference in the conventional biometric parameters (BPD, AC, FL, and EFW) between fetuses complicated with GDM and healthy pregnant women. Using the oral glucose tolerance test as the gold standard, we investigated the usefulness of measuring the thickness of the fetus's front abdominal wall as an early sonographic indicator of GDM. Our research found that using fetal AAWT as an early sonographic sign for diagnosing GDM while maintaining the oral glucose tolerance test as the gold standard yielded a sensitivity of 93.14, specificity of 82.65%, a positive predictive value of 92.05%, and a negative predictive value of 88.0%. According to Lertvutivivat S et al., measurement of AAWT in fetuses of 28 to 30 weeks pregnant women suffering from GDM has a sensitivity of 94.4% and specificity of 14.6%, taking 2 mm as the threshold for increased AAWT [5].

Previous research has shown that the increased fetal adiposity in GDM fetuses may be established as early as 20 weeks of pregnancy. The disparity between lean fetal mass and adiposity persists until 32 weeks of pregnancy [12-14]. Studies have shown that AAWT is much greater in women diagnosed with GDM despite other normal fetal biometrics being similar at the time of GDM screening. This has led researchers to speculate that AAWT may be an early ultra-sonographic indicator of GDM [6]. Prior research also showed that AAWT (>3.5, 4.5, and 5.5 mm at 30, 33, and 36 weeks of gestation, respectively) was more sensitive in diagnosing large for gestational age (LGA) than the use of abdominal circumference (AC) of >90th percentile [15]. According to the results of another research that only took a single measurement of AAWT, an AAWT of 5 mm between 28 and 34 weeks of gestation was the best predictor of macrosomia at term as measured by likelihood ratio. However, this cut-off had less sensitivity than an AC of >90th percentile [16]. Aksoy H et al. measured AAWT at the time of GDM screening and found that it was significantly different between GDM and control groups compared to standard biometry, which was similar in both groups. They commented that AAWT may have a role in evaluating fetal growth in GDM [6]. Another prospective clinical experiment looked at how regular measures of the fetal abdominal fat layer in the first few months of the third trimester could help treat diabetes pregnancies [16]. The researchers found that an abdominal fat layer in the fetus of 5 mm was the best predictor of macrosomia. They also mentioned that measuring the fetal belly fat layer should be routinely used to treat diabetes pregnancies but that further research is required to confirm this. Subcutaneous tissue thickness, including belly fat mass, was measured prospectively in both healthy and GDM pregnant women to establish normal ranges for fetal development [17]. In this analysis of pregnant women between 26 and 28 weeks of pregnancy, the median fetal abdominal fat mass was 3.58 mm. Recent prospective case-control clinical research matched 51 healthy pregnant women with 48 patients with impaired 50-g oral glucose tolerance tests but not GDM [18]. In pregnant women with compromised 50-g oral glucose tolerance tests during 26 and 28 weeks of gestation, the author observed substantially elevated fetal AAWTs. In addition, there was no difference between the damaged 50-g oral glucose tolerance tests and control groups in terms of fetal AC or EFW. Mean AAWTs were 4.05 + 0.51 and 3.69 + 0.50 in the study and healthy control groups, respectively (p <0.001).

## Conclusions

Measuring the fetus's AAWT is a straightforward procedure that may be performed alongside standard AC measurements. According to the results of this research, the thickness of the fetus's front abdominal wall may be used as an early sonographic indicator of GDM. Our findings suggest that measuring the thickness of the fetus' front abdominal wall may be an effective early sonographic indicator for identifying GDM and helping to avert the associated pregnancy problems.
